# Two Novel Precursors of the HIV-1 Protease Inhibitor Darunavir Target the UPR/Proteasome System in Human Hepatocellular Carcinoma Cell Line HepG2

**DOI:** 10.3390/cells10113052

**Published:** 2021-11-06

**Authors:** Roberta Rinaldi, Rocchina Miglionico, Ilaria Nigro, Rosarita D’Orsi, Lucia Chiummiento, Maria Funicello, Paolo Lupattelli, Ilaria Laurenzana, Alessandro Sgambato, Magnus Monné, Faustino Bisaccia, Maria Francesca Armentano

**Affiliations:** 1 Department of Sciences, Università degli Studi della Basilicata, 85100 Potenza, Italy; robertarinaldis3@gmail.com (R.R.); rocchina.miglionico@virgilio.it (R.M.); ilarianigro16@gmail.com (I.N.); rosarita.dorsi@gmail.com (R.D.); lucia.chiummiento@unibas.it (L.C.); maria.funicello@unibas.it (M.F.); paolo.lupattelli@unibas.it (P.L.); magnus.monne@unibas.it (M.M.); faustino.bisaccia@unibas.it (F.B.); 2 Laboratory of Preclinical and Translational Research, Centro di Riferimento Oncologico della Basilicata (IRCCS-CROB), 85028 Rionero in Vulture, Italy; ilaria.laurenzana@crob.it; 3 Scientific Direction, Centro di Riferimento Oncologico della Basilicata (IRCCS-CROB), 85028 Rionero in Vulture, Italy; alessandro.sgambato@crob.it

**Keywords:** HIV-1 protease inhibitors, apoptosis, ER stress, UPR, proteasome, autophagy, hydroxyethylamine derivatives, liver cancer

## Abstract

**Background:** Several pre-clinical and clinical reports suggest that HIV-1 protease inhibitors, in addition to the antiretroviral properties, possess pleiotropic pharmacological effects including anticancer action. Therefore, we investigated the pro-apoptotic activity in tumor cells of two molecules, RDD-19 and RDD-142, which are hydroxyethylamine derivatives’ precursors of darunavir and several HIV-1 protease inhibitors. **Methods**: Three hepatoma cell lines and one non-pathological cell line were treated with RDD-19 and RDD-142, and cell viability was assessed. The expression levels of several markers for ER stress, autophagy, cellular ubiquitination, and Akt activation were quantified in HepG2 cells treated with RDD-19 and RDD-142 to evaluate apoptotic and non-apoptotic cell death. **Results:** RDD-19 and RDD-142 showed a greater dose-dependent cytotoxicity towards the hepatic tumor cell line HepG2 compared to the non-pathological hepatic cell line IHH. Both molecules caused two types of cell death, a caspase-dependent apoptosis, which was ascertained by a series of biochemical and morphological assays, and a caspase-independent death that was characterized by the induction of ER stress and autophagy. The strong increase of ubiquitinated proteins inside the cells suggested that the target of these molecules could be the proteasome and in silico molecular docking analysis that was used to support the plausibility of this hypothesis. Furthermore, cells treated with the two compounds displayed decreased levels of p-AKT, which interferes with cell survival and proliferation. **Conclusions:** These findings demonstrate that two compounds, RDD-19 and RDD-142, have pleiotropic effects and that they may represent promising anticancer candidates.

## 1. Introduction

FDA-approved human immunodeficiency virus 1 protease inhibitors (HIV-PI) are used in highly active antiretroviral therapy (HAART) that significantly improves the clinical conditions of HIV patients [1,2]. To date, 10 small molecules, i.e., saquinavir, indinavir, ritonavir, nelfinavir, amprenavir, fosamprenavir, lopinavir, atazanavir, tipranavir and darunavir, all containing a mimicked peptide bond, have been designed to target the viral protease [3] and they are extensively used in the treatment of AIDS. Unexpectedly, in addition to the antiviral properties, several preclinical and clinical trials have highlighted pleiotropic effects of these molecules in malignancies not related to HIV, suggesting that they also have a pharmacological potential as antitumor agents in both virus- and non-virus-associated cancers [4,5]. In fact, HIV-PIs have been shown to counteract, among others, Kaposi sarcoma [6], multiple myeloma, breast cancer, and ovarian cancer [7,8,9,10,11,12,13,14] and to act as radio- and chemo-sensitizer [13,15,16,17,18]. Many mechanisms of action of HIV-PI in tumor cells have been proposed: interference with the cell cycle, triggering of both caspase-dependent and caspase-independent apoptotic pathways, causing oxidative stress and mitochondrial damage, inhibition of angiogenesis and tumor cell invasion [5]. In this study, we preliminarily performed viability assays on tumor and non-pathological cell lines to evaluate the cytotoxicity of a series of hydroxyethylamine derivatives, used as precursors of HIV-1 protease inhibitors and previously evaluated for their antiviral activity [19,20,21,22]. Subsequently, the two compounds most active on the hepatoma cell lines, RDD-19 and RDD-142, were used for more in-depth biological evaluations. In hepatic cancer cell lines both molecules similarly impair tumor cell viability by a caspase-dependent apoptotic mechanism but also through the induction of persistent and dose-dependent ER stress, which probably is caused by the inhibition of the proteasome, as suggested by the dose-dependent increase of poly-ubiquitinated proteins. Moreover, the prosurvival PI3K/Akt signaling activation pathway was reduced upon treatment with the two molecules. Taken together, our results suggest that RDD-19 and RDD-142 are able to reduce HepG2 cell survival through multiple mechanisms and may provide a starting point for further research aiming at their development into anticancer agents.

## 2. Materials and Methods

### 2.1. Chemicals and Antibodies

*N*-((2R,3S)-3-amino-2-hydroxy-4-phenylbutyl)-*N*-isobutyl-4-methoxybenzenesulfonamide and *N*-((2R,3S) -3-amino-2-hydroxy-4-phenylbutyl)-*N*-benzyl-4-methoxybenzenesulfonamide, indicated respectively with the abbreviations RDD-19 and RDD-142, were synthetized, purified, and finally characterized by NMR [19,20]. Dulbecco’s Modification of Eagle’s Medium was purchased from Corning. Dulbecco’s Modified Eagle’s Medium/Nutrient Mixture F-12 Ham, William’s E medium Dimethyl sulfoxide (DMSO), Trypsin–EDTA Solution, Thiazolyl Blue Tetrazolium Bromide (MTT), HOECHST 33258 Stain Solution, Doxorubicin hydrochloride, Bradford Reagent, and Darunavir were purchased from Sigma Aldrich-Merck. Dulbecco’s Phosphate Buffered Saline, *L*–Glutamine, Penicillin-Streptomycin Solution, and Fetal Bovine Serum were purchased from EuroClone. Primary antibodies specific for poly-ADP-ribose polymerase (PARP)(#9542, 116/89 kDa), LC3A/B (#12741, 16/14 kDa), CHOP (#2895, 27 kDa), IRE1α (#3294, 130 kDa), phospho-Akt(Ser473) (#4060, 60 kDa), Akt (#4691, 60 kDa), Ubiquitin (#3936), β-Actin (#3700, 45 kDa), Tubulin (#2144, 52 kDa), Anti-mouse IgG- HRP-linked (#7076), and Anti-rabbit IgG-HRP-linked (#7074) were purchased from Cell Signaling Technology (CST, Danvers, MA, USA). Primary antibodies specific for Beclin-1 (#849701, 52 kDa), sXBP1 (#647501, 55 kDa), ATF4 (#693901, 39 kDa), and ATF6 (#853101, 80 kDa) were purchased from Biolegend (San Diego, CA, USA). Primary antibodies specific for GRP78⁄ BiP (#ab21685, 75 kDa) and the anti-rat IgG-HRP-linked (#ab97057) were from Abcam. The primary antibody specific for p-PERK (#SAB5700521, 125 kDa) was purchased from Sigma Aldrich-Merck. The annexin V-PI apoptosis detection kit was purchased from BD Biosciences (San Jose, CA, USA).

### 2.2. Cell Lines and Culture Conditions

HepG2 and HuH7 (Hepatocellular carcinoma human cell lines) are immortalized human cells propagated in Dulbecco’s Modified Eagle Medium (DMEM) supplemented with 10% fetal bovine serum (FBS), 2 mM *L*-glutamine, and with 100 IU/mL penicillin/100 mg/mL streptomycin. JHH6, the third hepatocarcinoma cell line analyzed, was cultured in William’s E medium supplemented with 10% FBS, 2 mM *L*-glutamine, and 100 IU/mL penicillin/100 mg/mL streptomycin. IHH (Immortalized Human Hepatocytes) are human hepatocytes immortalized by stable transfection with the recombinant plasmid SV40 of healthy liver cells. They were propagated in Dulbecco’s Modified Eagle Medium/Nutrient Mixture F-12 supplemented with 10% FBS, 2 mM *L*-glutamine, 100 IU/mL penicillin/100 mg/mL streptomycin antibiotics, 1.2 × 10^−7^ M insulin from bovine pancreas, and 1 µM dexamethasone. All cells were cultured at 37 °C in a humidified, 5% CO_2_ atmosphere. HepG2 and Huh7 cells were kindly gifted by Dr. V. Infantino (University of Basilicata, Italy) and by Prof. G. Giannelli (University of Bari, Italy), respectively. JHH6 and IHH cells lines were kindly provided by Prof. C. Tiribelli (Liver research Center, Italian Liver Foundation, Trieste, Italy).

All the compounds were solubilized in dimethylsulfoxide (DMSO) as 50 mM stock solutions and stored at −20 °C until use. The working solutions were freshly prepared, diluting in cell culture medium: The final concentration of DMSO was always within the limit of 0.8% (*v*/*v*), which did not affect cell growth when compared with the vehicle-free controls.

### 2.3. Measurement of Cell Viability

Cytotoxicity of the synthetic molecules was assessed by MTT [3-(4,5-dimethylthiazol-2-yl)-2,5-diphenyltetrazolium bromide] colorimetric assay. Experimentally, HepG2, JHH6, HuH7, and IHH cells were seeded in 96-well plates (2 × 10^4^ cells per well) and incubated for 24 h. The cells were treated with different concentrations of molecules (150 µM, 100 µM, 75 µM, 50 µM, 37.5 µM, and 25 µM) for 24 h. As a negative control, cells were treated with DMSO at the highest concentration used in the treatments. Thereafter, the media were discarded and the cells were incubated with 100 µL of 0.75 mg/mL MTT solution for 4 h in the dark at 37 °C. Subsequently, formazan crystals were dissolved by the addition of 100 µL/well 1:1 DMSO:isopropanol. Absorbance was measured using a Multiskan Go spectrophotometer (Thermo Scientific, Waltham, MA, USA) at a wavelength of 570 nm with background subtraction at 630 nm. The 50% inhibitory concentration (IC50) was calculated using GraphPad Prism software (GraphPad, La Jolla, CA, USA).

### 2.4. Observation of Morphological Changes

HepG2 cells were seeded into 12-well plates (2 × 10^5^ cells per well) and cultured overnight. They were treated for 24 h with different concentrations of RDD-19 and RDD-142 (150 µM, 100 µM, 75 µM, 50 µM, 37.5 µM, and 25 µM), using untreated cells and cells treated with DMSO as negative controls. The cellular morphology was observed using phase contrast microscopy (Nikon Eclipse).

To underline the possible morphological changes of cells, such as cell fragmentation and chromatin condensation, cell cultures were labeled using the cell-permeable, DNA-specific fluorescent dye HOECHST 33258 (excitation at 352 nm, emission at 461 nm), according to [23]. Briefly, cells were seeded (2 × 10^5^ cells per well) in a 12-well plate, incubated at 37 °C with a 5% CO_2_ atmosphere and then treated with various concentrations of the molecules (75 µM, 50 µM, and 37.5 µM). Untreated cells and cells treated only with DMSO (vehicle) were used as negative controls. After 24-h incubation, cells were washed with phosphate buffered saline (PBS), fixed with 4% paraformaldehyde for 20 min at room temperature, then washed with pre-chilled PBS three times, and stained with approximately 10 µg/mL of HOECHST 33258 at room temperature in the dark for 10 min. The images were acquired using a fluorescence microscope (FLoid Cell Imaging Station, LIFE Technologies, Nikon 80i, Thermo Fisher Scientific, Waltham, MA, USA).

### 2.5. FACS Analysis

To assess the extent of apoptosis induction after 24 h of treatment with different concentrations (75 µM, 50 µM, and 37.5 µM) of RDD-19 and RDD-142, 5 × 10^5^ cells per well HepG2 cells were seeded in a six-well plate. As a positive control, cells were treated with 4.3 µM Doxorubicin. Untreated cells and cells treated with DMSO represented negative controls. After 24 h of treatment, cells were harvested, washed twice with cold PBS, and resuspended in Binding Buffer (FITC Annexin V Apoptosis Detection Kit I, BD Pharmigen, Franklin Lakes, NJ, USA). Then, 100 µL of the cell suspensions were mixed with 5 µL of Annexin V-FITC and 5 µL of propidium iodide (PI) and incubated in the dark for 15 min at room temperature. Then, 400 µL of Binding Buffer was added to each tube and the analysis was carried out by FACS (Navios, Beckman Coulter, IN, USA) with the Kaluza 2.1 analysis program (excitation at 488 nm, emission at 585 nm).

### 2.6. Western Blotting Analysis

HepG2 cells were seeded in six-well plates at 6 × 10^5^ cells per well and treated for 24 h at the concentrations of 75 µM, 50 µM, and 37.5 µM of the RDD 19 and RDD 142 inhibitors; then they were collected and lysed for 30 min in ice-cold RIPA buffer (Cell Signaling Technology, CST), supplemented with a mixture of PIC 100X (Phosphatase Inhibitor Cocktail—CST) and 1M PMSF (Phenylmethanesulfonyl Fluoride—CST). All the samples were also sonicated for 30 sec at 37% power with the BANDELIN SonoPuls sonicator and then centrifuged at 13,000 rpm for 20 min at 4 °C to remove insoluble cell debris. Protein content was quantified using the Bradford Reagent from Sigma. Equal amounts of protein were separated by SDS-PAGE and transferred onto nitrocellulose membrane. The membranes were blocked for 1 h with 5% nonfat dry milk in PBS and then overnight probed with the primary antibodies against BiP/GRP78 (1:1000), CHOP (1:1000), IRE1α (1:1000), sXBP1 (1:1000), ATF4 (1:1000), ATF6 (1:1000), PARP-1/cPARP-1 (1:1000), Beclin-1 (1:1000), LC3 A/B (1:1000), pAKT(Ser473) (1:1000), AKT (1:1000), ubiquitin (1:1000), p-PERK (1:1000), actin (1:1000), and tubulin (1:1000). The membranes were then washed three times with wash buffer (PBST), incubated with the appropriate horseradish peroxidase-linked secondary antibody (all diluted 1:3000), and visualized with the enhanced chemiluminescent detection system (ECL Star Enhanced Chemiluminescent Substrate, LiteAblot TURBO Extra Sensitive Chemiluminescent Substrate, from EuroClone, Milan, Italy). The chemiluminescent signal was detected through the Chemidoc XRS detection system (BioRad, Hercules, California, USA) with the ImageLab software and quantified by densitometric analysis of the bands using the ImageJ software 1.52a [24,25].

### 2.7. Quantitative RT-PCR for Nrf2 Gene Expression

HepG2 cells treated with different concentrations of RDD-19 and RDD-142 for 24 h were harvested and total RNA was extracted using the Quick-RNATM MiniPrep kit (Zymo Research), according to [26]. One microgram of RNA was used for cDNA preparation using the High-Capacity cDNA Reverse Transcription kit (Applied Biosystems, Waltham, Massachusetts, USA), according to the manufacturer’s instructions. The cDNA was amplified via Real-Time PCR using PowerSYBR Green PCR Master Mix (Promega) on the 7500 Fast Real-Time PCR System (Applied Biosystems). Primers, designed with the Allele ID program, spanned exon–exon junctions to eliminate any undesirable genomic DNA amplification. The cycling conditions were 95 °C for 10 min, followed by 40 cycles of 95 °C for 15 s, 60 °C for 60s, 72 °C for 90 s, and a final extension at 72 °C for 10 min. To confirm PCR specificity, the PCR products were subjected to a melting-curve analysis. The comparative threshold cycle method (ΔΔCt) was used to quantify relative amounts of product transcripts with β-actin as endogenous reference control. Primers set were: FOR: 5′-AACTACTCCCAGGTTGCCCA-3′ and REV: 5′-CATTGTCATCTACAAACGGGAA-3′.

### 2.8. In Silico Molecular Docking

Molecular docking was performed by AutoDock Vina [27] with the conformationally flexible compounds RDD-19 and RDD-142 into the structure of the human proteasome (PDB ID: 5LEY) [28]. The side chains of the residues in the inhibitor binding pockets (one between subunits β1–β5 and the other between β6–β7) were chosen to have conformational flexibility during the docking: the β 1–β5 site: chain L (β1): Y107, D125, S129, Q131, D133, and K136; chain K (β5): T1, D17, R19, S28, T21, V31, K33, M45, S53, S130, and Y169; and the β6–β7 site: chain N (β6): T1, I3, D17, R19, T20, T21, T22, N28, T31, D32, K33, R45, S46, S48, Q53, M95, M116, S130, D167, S169, and S170; chain H (β7): Y114, P115, H116, S118, and 120.

### 2.9. Statistical Analysis

All the results are presented as means ± Standard Error (SE) of three independent experiments performed in triplicate. The statistical significances were evaluated (GraphPad Prism version 8.4.2 for Windows, GraphPad Software, San Diego, CA, USA, www.graphpad.com, accessed on 8 April 2020) using one-way analysis of variance (ANOVA) followed by Dunnett’s post hoc test.

## 3. Results

**Figure 1:**
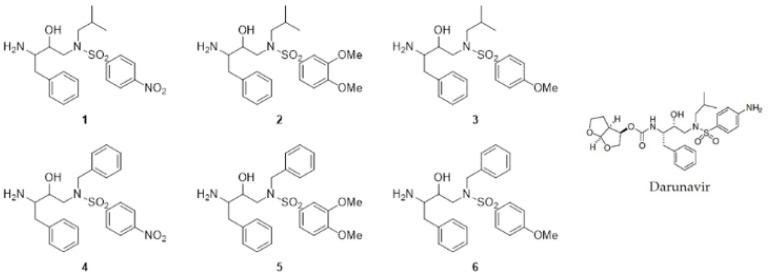
Chemical structures of compounds **1**–**6**, synthetized according to a general procedure [19,20], and Darunavir.

Compounds **1**–**6** (Figure 1), originally synthesized to be precursors of novel analogs of darunavir, were used for a preliminary viability assay using the non-tumor cell line IHH and hepatoma cell line HepG2. Our results showed that compounds **3** (later referred to as RDD-19) and **6** (later referred to as RDD-142) had a greater cytotoxic activity (IC50: 57.05 µM and 67.96 µM, respectively, as reported in Table 1) towards the tumor cell line than the other four molecules (IC50 > 150 µM in all cases) and for this reason they were selected for subsequent biological assays. Compounds **3** and **6** had a smaller steric hindrance of the sulfonyl than compounds **2** and **5** and a greater electron donor character of the sulfonyl substituents compared to compounds **1** and **4**. These structural differences could justify the different biological activity detected.

### 3.1. RDD-19 and RDD-142 Reduce Viability of Hepatocellular Carcinoma Lines in a Dose-Dependent Manner

**Figure 2:**
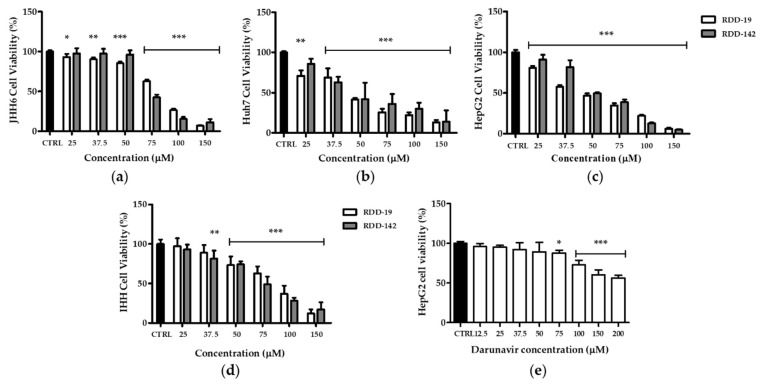
Cell viability assay. Cells were exposed to the indicated concentrations of RDD-19 and RDD-142 for 24 h and cell survival was measured using the MTT assay, as described in the Materials and Methods Section. RDD-19 and RDD-142 showed higher cytotoxicity to hepatoma cell lines (**a**–**c**) than a normal hepatic cell line (**d**). Darunavir treatment of HepG2 cells showed significantly lower cytotoxicity (**e**). The percentage of viable cells was calculated as the ratio of treated cells to control cells. Vehicle-treated cells were the negative control (CTRL). The data are expressed as means ± Standard Error (SE) of three replicates from three independent experiments and statistical significance was evaluated (GraphPad Prism 8.4.2 software) with one-way ANOVA followed by Dunnett’s post hoc test. Significance (* *p* < 0.05, ** *p* < 0.01, *** *p* < 0.001).

The cytotoxic effects of Darunavir, RDD-19, and RDD-142 to different hepatoma cell lines (HepG2, JHH6, and HuH7) and to a healthy hepatic cell line (IHH) were evaluated using the MTT assay. The assay was performed by treating cells for 24 h with different concentrations (150 µM, 100 µM, 75 µM, 50 µM, 37.5 µM, and 25 µM) of the molecules. As shown in Figure 2, both molecules showed greater cytotoxic effects in the three hepatocellular carcinoma lines (a,b,c) than in the non-tumor cell line (d). Since the cytotoxic effects of both molecules were higher overall towards the HepG2 cell line, it was chosen for all subsequent experiments. Cell viability of HepG2 cells treated with Darunavir was also evaluated (Figure 2e) and its poor cytotoxicity was revealed. All the IC50 values, calculated by linear regression, are reported in Table 1.

### 3.2. RDD-19 and RDD-142 Treatment Changes Cell Morphology of HepG2 Cells

**Figure 3:**
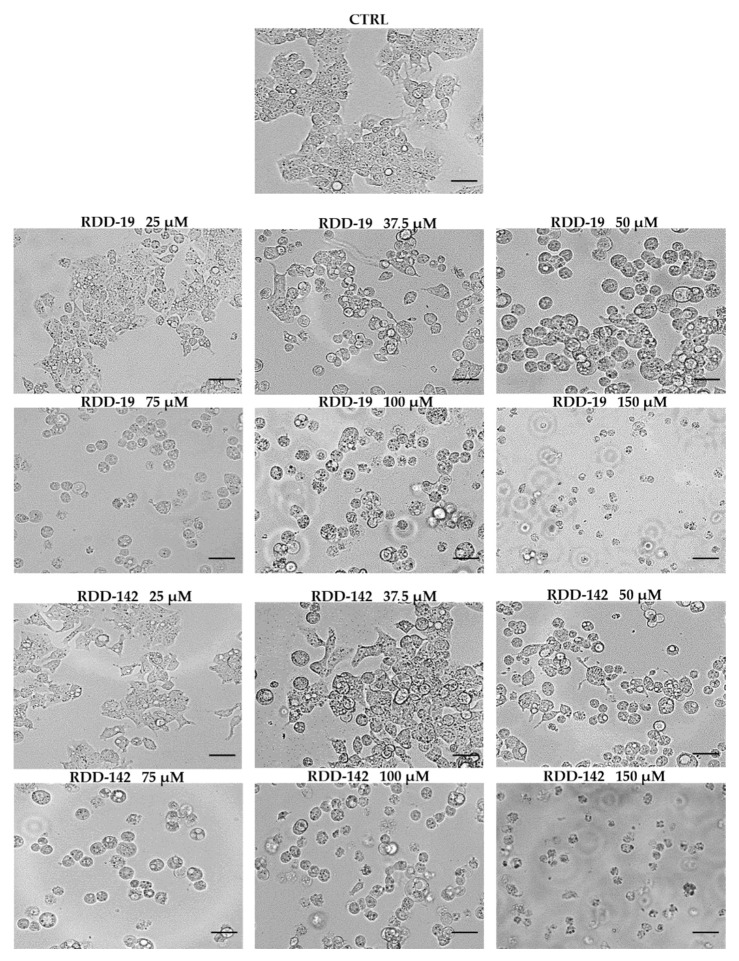
Morphological cell analysis. Inverted phase contrast images showing changes in HepG2 cells induced by different concentrations of RDD-19 and RDD-142. Vehicle-treated cells were the negative control (CTRL). Scale bars: 100 μm.

Morphological changes of HepG2 cells were detected after treatment with different concentrations of RDD-19 and RDD-142 for 24 h. Cell morphology was visualized by a light optical microscope (Nikon Eclipse TS100) with 40X magnification, as shown in Figure 3. The treatment of HepG2 cells with both molecules allowed us to appreciate a dose-dependent change in cell morphology: Already at 37.5 μM, the cells began to detach and to appear rounded, also showing intracellular vacuolation.

### 3.3. RDD-19 and RDD-142 Induce Apoptosis in HepG2 Cells

**Figure 4:**
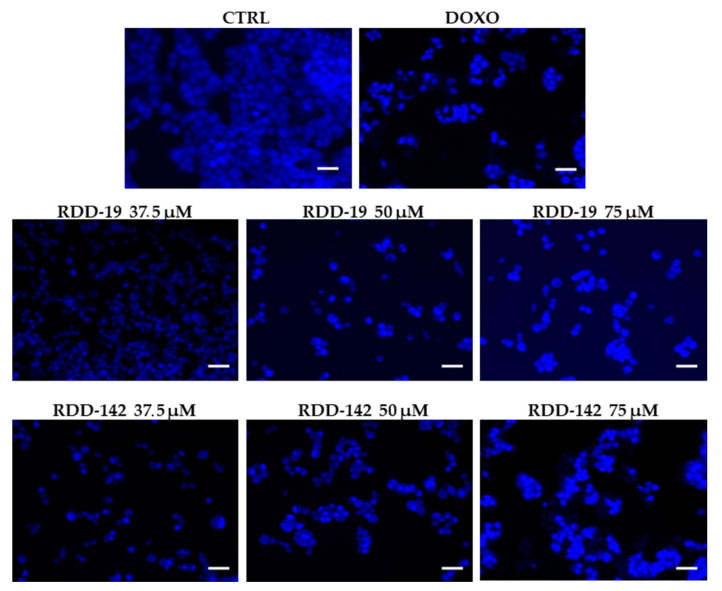
Qualitative analysis of apoptosis by Hoechst 33,258 staining. After the cells were treated with different concentrations of the RDD-19 and RDD-142, dose-dependent changes in nuclear morphology, such as condensation of chromatin, were observed under fluorescence microscope. Vehicle-treated cells and doxorubicin-treated cells were negative and positive controls, respectively. Scale bars: 50 μm.

A qualitative evaluation of a possible mechanism of apoptosis was carried out through the use of the distinctive apoptosis marker Hoechst 33,258 dye, which, by binding to DNA, allows the assessment of the condensation state of chromatin and/or nuclear morphology. As shown in Figure 4, the nuclear morphology in cells treated with vehicle DMSO (negative control) was of a regular shape and the staining was homogeneous and slightly diffuse. Both in the positive control (doxorubicin-treated cells) and in the molecules-treated cells we detected the accumulation of the fluorescent dye, in a dose-dependent manner, a sign of possible pyknosis and cell shrinkage. These findings suggested that treatment with our compounds could kill HepG2 cells through an apoptotic mechanism.

**Figure 5:**
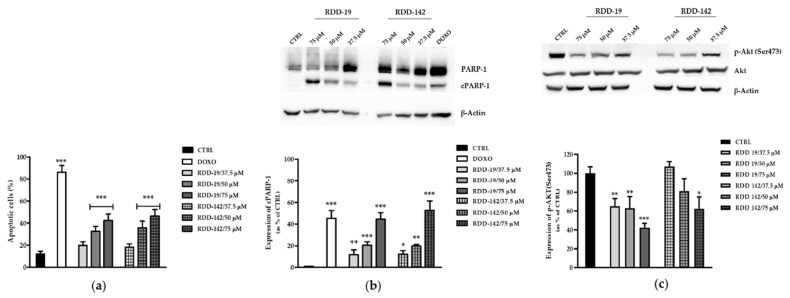
Quantitative analysis of apoptosis. (**a**) FACS analysis. The quantitative evaluation of apoptosis after 24 h of treatment of HepG2 cells with increasing concentrations of RDD-19 and RDD-142 showed a dose-dependent increase of the number of apoptotic cells. (**b**,**c**) Western blotting analysis. Cells’ treatment with the molecules induced apoptosis through intrinsic caspase-3/PARP-1 pathway, as demonstrated by the increased expression level of cPARP-1. PI3K/Akt survival pathway was also involved, as p-Akt(Ser473) expression levels were reduced. HepG2 cells were treated with DMSO as negative control (CTRL) and with doxorubicin as positive control. The data are expressed as means ± Standard Error (SE) of three replicates from three independent experiments and statistical significance was evaluated (GraphPad Prism software 8.4.2) with one-way ANOVA followed by Dunnett’s post hoc test. Significance (* *p* < 0.05, ** *p* < 0.01, *** *p* < 0.001).

To further confirm the induction of apoptosis, HepG2 cells were stained with annexin V-FITC and PI after treatment with different concentrations of the molecules for 24 h. The analyzed results show an increased number of Annexin V-FITC-positive cells, in a dose-dependent manner (Figure 5a). Furthermore, the comparison between the activities of the two molecules did not reveal any significant difference in their ability to induce apoptosis.

Finally, the expression of the nuclear protein poly(ADP-ribose)polymerase (PARP-1), an important substrate of activated caspase-3, and the PI3K/AKT pathway were evaluated. The PARP-1 cleavage is considered an important downstream signaling event considered as a hallmark of the apoptosis. Results reported in Figure 5b show clearly that it is present in a significant dose-dependent activation of caspase-3. Furthermore, the treatment with our compounds strongly affected the survival pathway PI3K/AKT, which is highlighted by a strong decrease in p-AKT(Ser473) expression, especially in RDD-19-treated cells (Figure 5c). These data allow us to state that the treatment of HepG2 cells with RDD-19 and RDD-142 molecules compromises cell survival by triggering apoptotic mechanisms. Future studies will ascertain a possible direct involvement of mitochondria, following the time- and dose-dependent treatment with the molecules, to better define the causes of cell death.

### 3.4. RDD-19 and RDD-142 Trigger UPR and Autophagy

**Figure 6:**
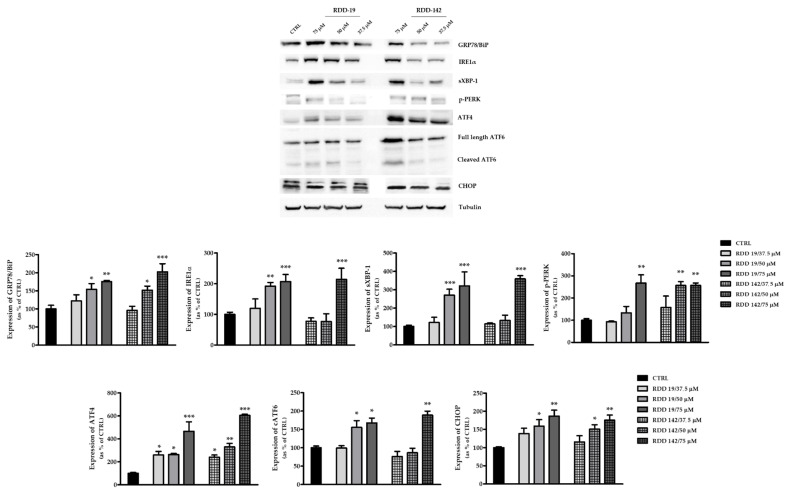
Western Blotting analysis of UPR-related proteins. Protein expression of biochemical markers of ER stress was evaluated by immunoblotting after 24 h of treatment of HepG2 cells with RDD-19 and RDD-142 at three different concentrations. HepG2 cells were treated with vehicle DMSO as negative control (CTRL). In the densitometric analysis, (Image J software 1.52a) results are expressed as percent of the control value. The data are expressed as means ± Standard Error (SE) of three replicates from three independent experiments and statistical significance was evaluated (GraphPad Prism software 8.4.2) with one-way ANOVA followed by Dunnett’s post hoc test. Significance (* *p* < 0.05, ** *p* < 0.01, *** *p* < 0.001).

It has been reported that several HIV-PI and their analogs have anticancer activity via induction of ER Stress (ERS) and autophagy and via the proteasome inhibition [7,9,18,29]. Thus, we sought to determine if our molecules affected HepG2 cells’ viability through one or more such mechanisms. The hepatic cancer cell line was treated with different concentrations of RDD-19 and RDD142, and the expression of specific ER stress markers was evaluated. As shown in Figure 6, the level of expression of the main ER stress marker, namely, the GRP78/BiP chaperone, was significantly altered, which suggests a strong activation of cellular Unfolded Protein Response (UPR).

To investigate further which of the three branches of the UPR was most affected, the expression levels of the main markers were assessed. The results suggest a certain activation of the IRE1a branch, given the increased protein expression of both IRE1a and sXBP1, which was more evident with RDD-19 (Figure 6). The PERK pathway was also activated, as evidenced by the increase in expression of p-PERK and ATF4. The expression of the pro-apoptotic transcription factor C/EBP homologous protein (CHOP) was also evaluated and, as shown in Figure 6, there was a dose-dependent increase in the amount of protein, which correlates both with the data on apoptosis shown above (Section 3.3) and with the upregulation of ATF4. Furthermore, the ATF6 branch also appeared to be activated following dose-dependent treatment with the two molecules.

**Figure 7:**
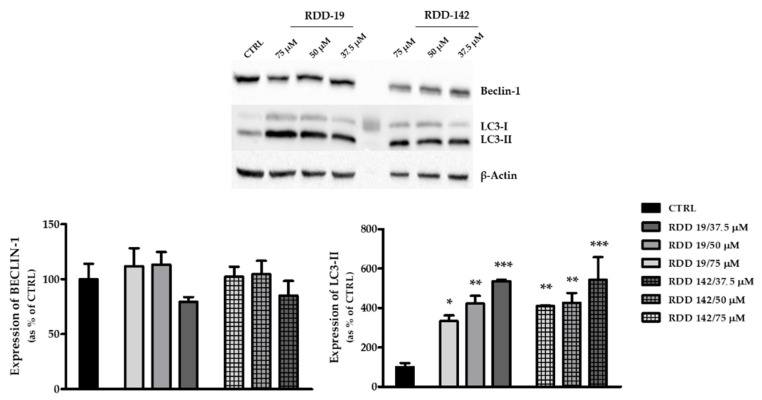
Evaluation of expression levels of autophagy-related proteins. Western blotting analysis of LC3-I, LC3-II, and Beclin-1 protein levels in HepG2 cells after 24 h of treatment of HepG2 cells with RDD-19 and RDD-142 at three different concentrations. β-Actin was used as an internal control. The intensity of the bands was quantified by densitometric analysis (Image J software 1.52a). The data are expressed as means ± Standard Error (SE) of three replicates from three independent experiments and statistical significance was evaluated (GraphPad Prism software 8.4.2) with one-way ANOVA followed by Dunnett’s post hoc test. Significance (* *p* < 0.05, ** *p* < 0.01, *** *p* < 0.001).

It is well known that the condition of cellular ER stress is related to the activation of autophagy processes [30]. For this reason, the expression levels of two important markers of this mechanism were evaluated, namely, the microtubule-associated protein 1A⁄1B-light chain 3 (LC3), which exists as a cytoplasmic form (LC3-I) or as a membranous form (LC3-II), which is a component of autophagosomes, and the protein Beclin-1, an important regulator of early-stage autophagy. After treatment with RDD-19 and RDD-142, a significant increase in the expression of LC3-II was detected using both the compounds, while the expression of Beclin-1 seems to remain unchanged (Figure 7). The results suggest that there are caspase-independent mechanisms activated following treatment with the synthetic precursors.

### 3.5. Inhibition of the Proteasome by RDD-19 and RDD-142

**Figure 8:**
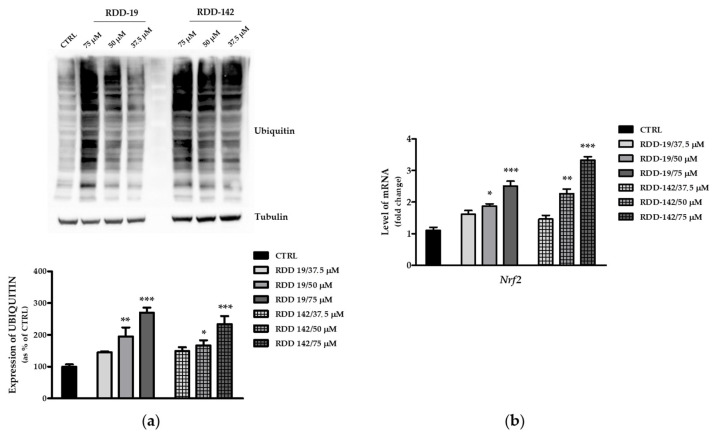
Proteasome inhibition assessment. (**a**) Representative immunoblot of total ubiquitinated proteins from HepG2 cells treated with three different concentrations of RDD-19 and RDD-142. Tubulin was used as loading control and cells treated with vehicle DMSO were used as negative control. In the densitometric analysis, results are expressed as percent of the control value. (**b**) Quantification of Nrf2 target transcript in Real Time qPCR, measured in HepG2 cells treated with our precursors. The data are expressed as means ± Standard Error (SE) of three replicates from three independent experiments and statistical significance was evaluated (GraphPad Prism software 8.4.2) with one-way ANOVA followed by Dunnett’s post hoc test. Significance (* *p* < 0.05, ** *p* < 0.01, *** *p* < 0.001).

Some studies reported the ability of HIV-PIs and some of their analogs to inhibit the proteasome [3,7,18]. In this paper, we assessed this possibility of measuring the level of ubiquitination of cellular proteins, considered as a surrogate marker of proteasome inhibition. As shown in Figure 8a, treatment with RDD-19 and RDD-142 resulted in the notable increase in protein ubiquitination.

The hypothesis of proteasome inhibition was further investigated by evaluating the gene expression level of Nrf2, one of the transcription factors involved in the de novo biosynthesis of the proteasome subunits. The qPCR data, shown in Figure 8b, confirmed the dose-dependent increase in expression of this transcription factor, strengthening the hypothesis of an effective inhibition of the proteasome.

### 3.6. Molecular Docking of RDD-19 and RDD-142 in Inhibitor-Binding Sites on the Proteasome

Molecular docking was performed to analyze the potential binding capabilities of RDD-19 and RDD-142 in already identified inhibitor-binding sites in the structure of the human proteasome. In the docking procedure, the side chains of the residues of the inhibitor-binding pocket and the tested ligand compounds are given conformational freedom, and solutions with the most favorable binding energies (highest affinities) are calculated upon searches in the so-called conformational space.

**Figure 9:**
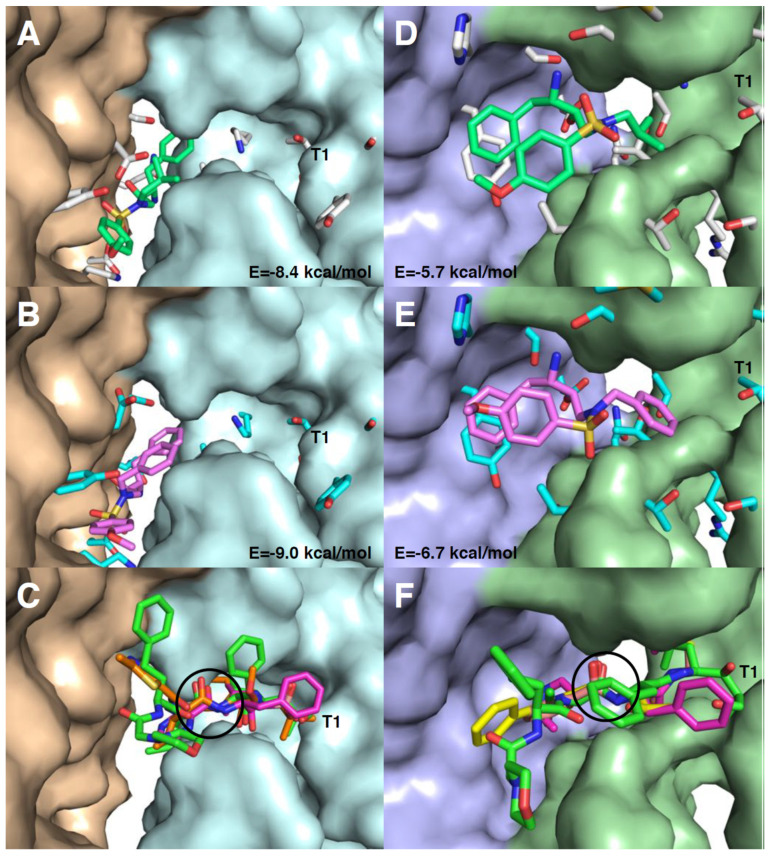
RDD-19 and RDD-142 docked into the structure of the human proteasome. The highest ranked docking solutions RDD-19 (sticks with carbons in lime-green; (**A**,**D**)) and RDD-142 (violet; (**B**,**E**)) surrounded by the flexible side chains (sticks with carbons in white (**A**,**D**) or cyan (**B**,**E**)) in the inhibitor-binding sites between subunits β1–β5 (wheat-pale cyan surfaces in (**A–C**)) and β6–β7 (pale green-light blue surfaces in (**D**–**F**)). The binding energies of the docking solutions are indicated in kcal/mol. The known proteasome inhibitors, which bind in these two sites of the proteasome, are shown in (**C**,**F**): carfilzomib (green carbons), bortezomib (magenta), delanzomib (yellow), ixazomib (salmon), and oprozomib (orange) with the minimal ligand structure in common (in the same position) encircled (a peptide bond or similar to a peptide bond). In all panels, T1 indicates threonine-1 that is linked covalently by the inhibitors in (**C**,**F**).

The highest-ranking docking results of RDD-19 and RDD-142 in the two known inhibitor binding sites at the interface between subunits β1–β5 and that between β6–β7 in the proteasome were compared to the binding of carfilzomib, bortezomib, delanzomib, ixazomib, and oprozomib, which are present in various 3D structures (Figure 9) [28]. In the β1–β5 inhibitor-binding site, RDD-19 and RDD-142 took similar poses and they protruded deep into the cleft in between the β1 and β5 subunits (Figure 9A,B), which is quite different from the position of carfilzomib, bortezomib, and oprozomib (Figure 9C), which are covalently linked to threonine-1 of subunit β5 after reaction with their epoxyketone and boronic acid groups. The docking solutions of RDD-19 and RDD-142 in the β1–β5 inhibitor-binding site exhibited quite high binding energies (−8.4 and −9.0 kcal/mol, respectively) compared to the same compounds in the β6–β7 inhibitor-binding site (−5.7 and −6.7 kcal/mol, respectively). Additionally, in the β6–β7 inhibitor-binding site, RDD-19 and RDD-142 had similar conformations and localizations in the binding pocket (Figure 9D,E); however, in this case, their position overlapped with those of carfilzomib, bortezomib, delanzomib, and ixazomib (Figure 9F), which were covalently linked to threonine-1 of the β6 subunit. In conclusion, according to the in silico docking results, RDD-19 and RDD-142 are compatible with binding the β1–β5 and/or β6–β7 inhibitor-binding sites with quite high binding affinities and in similar orientations and positions in each site, and, in the latter interface, their location overlapped with known proteasome inhibitors.

## 4. Discussion

The introduction of HAART significantly decreased the incidence of several forms of cancer, previously frequent in HIV-positive patients [31]. The effects of these drugs cannot be attributed entirely to their ability to suppress viral replication that restores almost normal immune function, but to direct anticancer effects, which are observed also in HIV-negative patients and related to their specific action as inhibitors of angiogenesis and tumor growth and progression, and as inducers of apoptosis [32]. The effects of HAART on tumor incidence and regression were more evident in studies involving patients treated with viral protease inhibitors (PI-HAART) than with viral reverse transcriptase inhibitors (NNRTI-HAART), which suggests unexpected specific anticancer effects of the PIs. Hence, many studies have been concerned with the evaluation of these non-antiviral actions of PIs (mainly of ritonavir, saquinavir, indinavir, and nelfinavir), ascertaining that, with their pleiotropic effects, HIV-PIs directly influence pathways involved in tumor cell survival and proliferation, angiogenesis, invasion, and inflammation [5,33].

The effects of HIV-PIs on liver cancer are less characterized than other types of cancer, despite that hepatocellular carcinoma (HCC) is one of the most widespread and growing diseases in the world and it is the most common form of liver cancer, accounting for 90% of the cases [34]. Its common risk factors are HBV/HCV infection, cirrhosis, NASH, metabolic syndrome, and diabetes mellitus. Surgery, percutaneous ablation, and liver transplantation are, at present, the preferred, but drastic, therapeutic options for HCC patients. However, in the last 10 years significant improvement has been made with pharmacological treatments in early-stage patients, which are now also applied to the majority of patients in intermediate and advanced stages. Systemic drug treatment involves the use of Immune-checkpoint Inhibitors (ICIs), Tyrosine Kinase Inhibitors (TKI), and monoclonal antibodies [34,35]. Additionally, the traditional chemotherapies that have been employed in the treatment of HCC have been shown to be inefficient to drastically improve the outcomes. Therefore, the identification of potential therapeutic targets, derived from the understanding of the molecular alterations of HCC, and of new drugs with a high therapeutic index targeting them are crucial for the future development of novel and improved HCC treatments.

Cancer cells, compared to non-tumorigenic cells, are characterized by fast metabolism and proliferation, which are accompanied by an aberrant production, folding, degradation, and turnover of proteins. In this regard, the Unfolded Protein Response (UPR) triggered in the ER and the protein degradation mechanisms, mediated by the ubiquitin–proteasome pathway, play a key role in the regulation of cell fate [36]. Therefore, the UPR/proteasome system is a rational target for therapeutic strategies aimed at shifting the protein turnover equilibrium towards the induction of tumor cell death [37]. Two therapeutic strategies for the treatment of cancer could, therefore, be as follows: (1) to inhibit the inducible UPR, which represents a cell survival mechanism and/or (2) to increase the ER stress state beyond the adaptive limit, overwhelming the UPR capacity and triggering apoptotic mechanisms [38].

Several molecules have been developed and tested (and some of these have been approved by the FDA) as modulators of the UPR/degradation machinery and they have been shown to be particularly effective on hematological malignancies [37,39,40]. Among these, some proteasome inhibitors have been examined as potential candidates also in HCC therapy [36,41,42,43,44], either alone or in combination with other therapies against HCC; while some preclinical studies showed promising results, unfortunately, clinical tests have been less encouraging. In fact, it is known that current FDA-approved proteasome inhibitors (bortezomib, carfilzomib, and ixazomib) have a limited effect on solid tumors [45] and a proposed mechanism to account for the resistance of the cancer cells for these drugs involves the pharmacokinetic and the pharmacodynamic properties of specific tested inhibitors. The conclusions from these studies, therefore, suggest that testing of new alternative small molecule proteasome inhibitors is warranted.

In the present study, the antitumor activity of two synthetic intermediates of darunavir analogs, RDD-19 and RDD-142, was evaluated. We considered a panel of three liver tumor cell lines, characterized by different degrees of differentiation (HepG2, HuH7, and JHH6 were assigned to high, medium, and low hepatocytic differentiation, respectively), and an immortalized non-pathological liver cell line IHH. Our results showed a phenotype-dependent cytotoxicity, being greater for the more differentiated cell lines HepG2 and HuH7 than for JHH6, as already reported in the literature for another proteasome inhibitor [46]. This led us to hypothesize a future experimentation of the molecules in animal models with different forms of HCC; however, the HepG2 hepatoma cell line was then chosen for subsequent in vitro investigations. The IC50 values shown could be considered suboptimal; however, it is worth emphasizing that RDD-19 and RDD-142 must be considered as promising lead compounds for the future development of, hopefully, more specific and effective molecules. For this purpose, the preliminary IC50 values obtained using their formulation in nanoparticles, capable of giving the molecules a greater possibility of cellular penetration than the unformulated molecules (data not yet published) are very promising. Furthermore, it is also conceivable to test them in combination with other drugs capable of acting with different mechanisms, for a more effective overall therapy. The two compounds caused cell death prevalently by a dose-dependent induction of apoptosis, as shown by FACS analysis, and further supported by the observation of the increased levels of the cleaved form of PARP as a result of caspase-3 activation. RDD-19 and RDD-142 treatment additionally compromised cell survival by the dose-dependent decrease of p-AKT. The possible involvement of the proteasome and the ER in the cell death induced by the two substances was examined because several HIV-PIs have been reported to act as proteasome inhibitors and in exacerbating ER stress [7,18]. The expression levels of different ER stress markers were measured to determine which of the three UPR pathways was implicated in the response to the treatment. Our results suggest that the molecules RDD-19 and RDD-142 were both able to activate all three branches of the UPR because of a strong ER stress state, which was observed by the upregulation of the representative ER-resident chaperone GRP78/BiP. As a result of the treatments with our two darunavir analogs’ precursors, the PERK branch was strongly activated, as indicated by the increase of its phosphorylated form. Furthermore, both substances increased expression levels of ATF4, and the pro-apoptotic transcription factor CHOP was detected, which corroborated the results on apoptosis discussed above [47]. The ATF6 branch was slightly activated, allowing the transcriptional regulation of genes encoding ER chaperones and enzymes that promote ER protein translocation, folding, maturation, and secretion as well as degradation of misfolded proteins. However, although the UPR was activated, apparently it was unable to maintain homeostasis and the proteotoxic state triggered apoptotic cell death.

The IRE1a branch was also activated, as confirmed by the upregulation of sXBP1, a transcription factor responsible for the expression of chaperones and genes involved in the ERAD system, which is capable of providing for the disposal of unfolded and misfolded proteins from the ER. Due to the high quantity of ubiquitinated proteins present after treatment with both compounds, we would like to hypothesize that an inhibition at the level of the proteasome occurred, which hindered the regular disposal of the proteins coming from the ER and thus creating a proteotoxic stress.

Several studies [36,48,49] have shown that treatment with proteasome inhibitors, routinely used in clinical settings, allows for increased expression levels of LC3-II, a classic marker of autophagosome formation, which is explained as an attempt by cells to reduce stress and restore normal cellular proteostasis. In RDD-19- and RDD-142-treated cells, we found an increased expression of LC3-II, which further indicated a real inhibition of the proteasome. Moreover, our results showed the increased expression of Nrf2, which is one of the transcription factors important for the de novo synthesis of 20S proteasome subunits and it is activated precisely in response to proteasome inhibition for increased proteasome biogenesis [49,50].

The molecular docking studies of RDD-19 and RDD-142 in the human proteasome demonstrated the potential of the two molecules to bind in two of the binding pockets for known proteasome inhibitors. It is noteworthy that the two compounds, which are structurally very similar, both bind to each of the two binding sites in a similar position and conformation, which gives credibility to the docking solutions. It is also interesting that, whereas the two molecules bind in a similar way to the known proteasome inhibitors in the interface between the β6–β7 subunits, in the β1–β5 interface they are binding in a totally different way. It should be mentioned that another HIV protease inhibitor, ritonavir, which is structurally quite different from darunavir, has been found to inhibit the proteasome and, based on docking studies, that it is binding one of the inhibitor binding pockets of the yeast proteasome [51], in a similar scenario to what is suggested for RDD-19 and RDD-142. Unlike the previously identified proteasome inhibitors that all seem to bind covalently to the threonine-1 of the proteasome subunits through a reaction with their reactive groups, ritonavir, RDD-19, and RDD-142, which lack reactivity, could interact with the proteasome without forming covalent bonds, suggesting that their inhibition is more easily reversible compared to the other inhibitors. This property of RDD-19 and RDD-142 may be a pharmacodynamic advantage in the future therapeutic use of these molecules as potential proteasome inhibitors.

Darunavir is a second-generation HIV-PI, highly active against the HIV-1 protease but with less frequent adverse effects. Some pleiotropic effects of darunavir are known, e.g., being able to prevent kidney injury via HIV-independent mechanisms [44] or showing anti-inflammatory and intestinal-protective properties [45]. Prior to our results, few studies had been performed on the anticancer properties of darunavir and/or its analogues. Its antitumor activity was evaluated in the PEL (Primary Effusion Lymphoma) cell line, showing no cytotoxic and pro-apoptotic effects [47]. Another study reported in silico evaluations of darunavir analogs as anti-cancer agents against five tumor lines (bone, brain, breast, colon, and skin cancer) and suggested that they are plausible candidates for continuing in vitro and in vivo testing [48]. Therefore, the present study is the first that analyzed the anticancer properties, on hepatoma cell lines, of two precursors of darunavir analogues containing an hydroxyethylamine core linked to *N*-isobutyl or *N*-benzyl p-methoxyphenylsulfonamide and a free primary amine. Our results suggest a plausible mechanism of action that is consistent with the literature present for other HIV-PIs. Considering that FDA-approved proteasome inhibitors have several side effects in patients [52] and show limited efficacy on solid tumors, the discovery and development of new drug therapies is essential. Our results indicate that RDD-19 and RDD-142 are to be considered promising candidates to deal with HCC, which justifies further investigation and clinical validation.

## Figures and Tables

**Table 1:** Half-maximal concentrations of RDD-19 and RDD-142 for inhibition of human hepatocarcinoma cell line viability.

Cell Line	IC50 (µM)		
	RDD-19	RDD-142	Darunavir
IHH	106.9	99.02	-
JHH6	96.61	95.04	-
HuH7	55.52	75.10	-
HepG2	57.05	67.96	>200
